# Microbiome Big-Data Mining and Applications Using Single-Cell Technologies and Metagenomics Approaches Toward Precision Medicine

**DOI:** 10.3389/fgene.2019.00972

**Published:** 2019-10-09

**Authors:** Mingyue Cheng, Le Cao, Kang Ning

**Affiliations:** Key Laboratory of Molecular Biophysics of the Ministry of Education, Hubei Key Laboratory of Bioinformatics and Molecular Imaging, Department of Bioinformatics and Systems Biology, College of Life Science and Technology, Huazhong University of Science and Technology, Wuhan, China

**Keywords:** big data, microbiome, metagenomics, single-cell sequencing, precision medicine

## Abstract

With the development of high-throughput sequencing technologies as well as various bioinformatics analytic tools, microbiome is not a “microbial dark matter” anymore. In this review, we first summarized the current analytical strategies used for big-data mining such as single-cell sequencing and metagenomics. We then provided insights into the integration of these strategies, showing significant advantages in fully describing microbiome from multiple aspects. Moreover, we discussed the correlation between gut microbiome with host organs and diseases, confirming the importance of big-data mining in clinical practices. We finally proposed new ideas about the trend of big-data mining in microbiome using multi-omics approaches and single-cell sequencing. The integration of multi-omics approaches and single-cell sequencing can provide full understanding of microbiome at both macroscopic level and microscopic level, thus contributing to precision medicine.

## Strategies for Big-Data Mining

The human gut microbiome has been confirmed to highly correlate with human health and diseases, through influencing human metabolism, nutrition, physiology, and immune function (Hooper and Gordon, 2001; Bäckhed et al., 2005; Manichanh et al., 2012). Hence, the characterization of the human gut microbiome, as well as its correlation with diseases, has fascinated a great number of researchers to explore. However, the human gut microbiome consists of approximately 15,000 to 36,000 species of bacteria (Frank et al., 2007), with the total number of bacterial cells ranging from 10^13^ to 10^14^, which is of the same order as the number of human cells (3.0 × 10^13^) (Sender et al., 2016). The gut microbiome also contains more than 100 times more genes, compared with 25,000 genes in humans (Gill et al., 2006). Considering this big data of the gut microbiome, sequencing would be a promising technology for mining it, rather than the traditional cultural methods. Sequencing is the precondition for obtaining raw genetic materials of the gut microbiome, followed by genetic assembly and taxonomic and functional annotations. Several strategies are currently used for big-data mining in microbial communities from different perspectives as follows (**Table 1**).

**Table 1:** The overview of pros and cons of current widely used methods for dissecting microbiome.

Methods	Advantages	Disadvantages	Solution
Amplicon sequencing	(1) Relatively low cost; (2) Taxonomic annotations of uncultured microbial communities.	(1) Low resolution: cannot identify microbes at species or stain level; (2) Cannot realize functional annotations of microbial communities.	(1) Combined with metagenomics; (2) Use PICRUSt to obtain predicted metagenomics and functional annotations.
Metagenomic sequencing	(1) Taxonomic and functional annotations of uncultured microbial communities; (2) Obtain the full genetic repertoire of the microbial communities.	(1) Difficulties in metagenome assembly and taxonomically and functionally assign accurately; (2) Lack of high genome coverage; (3) Cannot link all the functional genes of one microbe to its phylogeny.	(1) Long-read sequencing and improved algorithms for assembly; (2) Combined with single-cell sequencing.
Single-cell sequencing	(1) Taxonomic and functional annotations of uncultured microbes at cell level; (2) Generate a high-quality genome for microbes with low abundance; (3) Dissect virus-host interactions of uncultured microbes.	(1) Difficulties in cell sorting; (2) Easily influenced by contaminated DNA; (3) Uneven read coverage, chimeric reads caused by MDA.	(1) Combined with metagenomics; (2) Improved experimental operation and various computational approaches to control DNA contamination and errors caused by MDA.

### Amplicon Sequencing

Amplicon sequencing uses specific marker genes of microbes such as 16S ribosomal RNA for bacteria and Internal Transcribed Spacer (ITS) for fungi. This sequencing method mainly answers “who is there” in an uncultured microbial community by assigning reads to reference reads. However, low-resolution level (cannot reach to species or strain level) of amplicon sequencing, as well as its disability in functional annotation, largely limits its application. Therefore, current solution for this problem is to combine the amplicon sequencing and the metagenomic sequencing. Researchers can first use relatively low-cost amplicon sequencing to have a preliminary understanding of the composition of the targeted microbial community, thus determining the hypothesis. Subsequently, they can perform metagenomic sequencing to confirm the hypothesis from a perspective of both phylogeny and functions.

### Metagenomic Sequencing

The shotgun metagenomic sequencing process consists of DNA extraction from all cells in a community, DNA fragmentation, DNA sequencing, and sequence analysis such as marker gene analysis, binning, or contig assembly to obtain the taxonomic composition. Metagenomic sequencing not only can shed light on “who is there” at a high resolution to strain level, but also “what are they doing.” The metagenomic reads encoding proteins can be predicted for functional annotation, through various ways including gene fragment recruitment, protein family classification, and *de novo* gene prediction (Sharpton, 2014). The disadvantages of metagenomics sequencing are as follows. First, there are limitations of short reads produced by next-generation sequencing and the complexity in sequence assembly, especially when multiple strains are present (Sczyrba et al., 2017). For instance, the closely related genomes in a community might represent genome-sized approximate repeats. Second, metagenomic sequencing cannot obtain high genome coverage and might even lose genomes of low abundant microbes, owing to the high genomic richness and evenness in a community (Mende et al., 2016). Third, functional genes of one microbe cannot be fully linked to its phylogeny. There are two solutions for these problems. First, long-read sequencing can solve the ambiguity in sequence assembly (Bertrand et al., 2019). A recent method named OPERA-MS (Bertrand et al., 2019), which combines nanopore-sequenced long reads and Illumina-sequenced short reads through a hybrid metagenomic assembler, succeeds to promote the accuracy of strain-resolved assembly and obtains genomes with higher coverage. The second solution is to combine metagenomics with single-cell sequencing, which can reconstruct how DNA is compartmentalized into cells and link functions to their corresponding species (Tolonen and Xavier, 2017).

### Single-Cell Sequencing

The first step of single-cell sequencing is to isolate the individual cells, using serial dilution, microfluidics, flow cytometry, micromanipulation, or encapsulation in droplets (Bäckhed et al., 2005). The following steps include DNA extraction, whole-genome amplification, DNA sequencing, and sequence analysis such as alignment and assembly. Owing to the fact that minimum requirement of high-throughput sequencing is micrograms, which is more than the femtograms of DNA a bacterial cell generally contains, amplification of the minute amounts of DNA of the cell is necessary (Xu and Zhao, 2018). For this purpose, a non–polymerase chain reaction–based DNA amplification method multiple displacement amplification (MDA) (Dean et al., 2002) uses random hexamer primers annealed to the template and a high-fidelity polymerase of the *Bacillus subtilis* phage phi29 (Blanco et al., 1989). The Phi29 DNA polymerase can work at a moderate isothermal condition, with a high-strand displacement activity and an inherent 3′–5′ proofreading exonuclease activity, thus ensuring enough genome coverage with lower amplification error for the following sequencing analysis.

The major advantage of single-cell sequencing is that it can generate a high-quality genome for species with low abundance, which might be lost by the metagenomic sequencing. Additionally, this method can discriminate and validate the functions of individuals within the community, linking these functions to specific species. Moreover, the single-cell sequencing can simultaneously recover bacterial genomes and extrachromosomal genetic materials in a cell, dissecting virus–host interactions at cell level (Yoon et al., 2011). Single-cell sequencing has already led to many novel findings such as the discovery of bacteria with an alternative genetic code (Campbell et al., 2013), the ability to observe which gut microbial cells use host-derived compounds (Berry et al., 2013), and the ability to quantify the absolute taxon abundances of the gut microbiome (Props et al., 2017).

However, the single-cell sequencing also has limitations as follows. First, cell sorting is a complicated and time-consuming process. Isolating cells from solid medium such as swabs, biopsies, and tissues remains challenging (Tolonen and Xavier, 2017). Second, the amplification step using MDA might magnify the DNA contamination. DNA contamination is mainly from the tainted specimen at the step of cell sorting, polluted reagents or laboratory apparatuses, and microbes in the environment. The solution for the contamination is to keep strictly clean of the work area with extra precaution. In addition, the reaction volume can be moderately reduced to increase the ratio of targeted DNA to the contaminated DNA. Moreover, contaminated DNA can be partly removed by aligning the reads to the reference of potentially contaminated DNA of human and environment. The third limitation is that the MDA procedure would cause highly uneven read coverage and increased formation of chimera reads that links nonadjacent template sequences; thus, conventional genome-assembly algorithms are not suitable for single-cell data. The solution for uneven read coverage is to normalize the reads by trimming the reads according to their k-mer depth, which has been integrated to several assembly algorithms such as SPAdes (Bankevich et al., 2012). The solution for chimera reads is to identify and remove the chimeras. Owing to the lack of reference genome of a certain number of cells, metagenomic sequencing can provide the contigs as reference for identifying chimeras.

### The Integration of Single-Cell Genomics and Metagenomics

**Figure 1:**
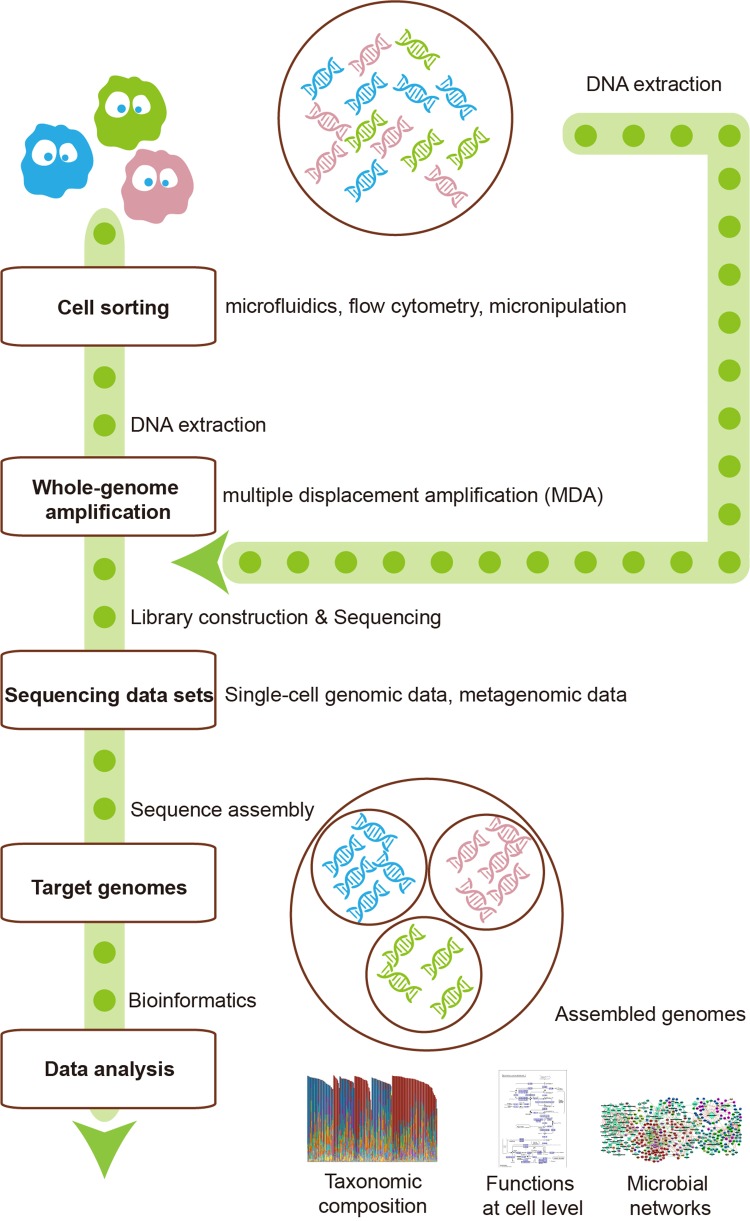
The integration of single-cell sequencing and metagenomics makes them complement each other. Single-cell sequencing could provide metagenomics with reference scaffolds, while metagenomics could ameliorate the genome assembly of single-cell sequencing.

The metagenomics represents the whole genome of all microbes in the environment, while single-cell genomics refers to the genomes of individuals cells that may or may not contain the full genetic repertoire in the microbiota. Hence, the integration of these two technologies can make up for each other’s shortcomings (**Figure 1**). For instance, reads and contigs of metagenomics can improve the genome assembly of single-cell genomics (Mende et al., 2016). Conversely, single-cell genomics can serve as scaffolds for comparison or recruitment of metagenomics when reference genomes are unavailable (Swan et al., 2013; Roux et al., 2014). Several studies have generated much-improved microbe genome assemblies from a variety of microbial communities, using the integration of single-cell genomics and metagenomics (Dupont et al., 2012; Nobu et al., 2015). The disadvantage of this integration is that the potential errors of both methods would be gathered, thus requiring more sophisticated methods to deal with.

### The Integration of Metagenomics and Three-Dimensional Genomics

Metagenomics can quantify the genetic materials of a microbial community, while the Hi-C sequencing can identify all chromatin interactions of the community, producing three-dimensional (3D) genome, reflecting both the genetic content and topological chromatin structures into digital information (Belaghzal et al., 2017). The integration of metagenomics and 3D genomics can fully display the composition and structure of genomes of a microbial community. Moreover, a recent study performed Hi-C for single-cell analysis, to capture 3D genomes of individual cells (Nagano et al., 2017).

### Microbial Multi-Omics Analysis

With advances in high-throughput sequencing technologies and bioinformatics approaches, researchers are now able to perform comprehensive analysis in microbial communities, named as “multi-omics analysis.” This analysis integrates metagenome, metatranscriptome, metaproteome, and metabolome. The metagenome displays the taxonomic composition in a microbial community and predicted functional expression. The metatranscriptome, metaproteome, and metabolome can confirm the predicted functions, further unveiling how microbes work in a community. These omics can provide significant information about a microbial community from different perspectives. For instance, the microbial communities of twins with Crohn disease have been analyzed at phylogenetic, functional, and metabolic levels, using 16S sequencing (Dicksved et al., 2008; Willing et al., 2009; Willing et al., 2010), metagenomics, proteomics (Erickson et al., 2012), and metabolomics (Jansson et al., 2009).The subjects with Crohn disease contain a microbial community with lower microbial diversity, depletion of *Faecalibacterium prausnitzii*, and lower expression levels of proteins involved in butyrate metabolism (Erickson et al., 2012). At the metabolite level, thousands of metabolites such as the bile acids (BAs) that were detected higher in diseased subjects can distinguish healthy subjects from subjects with Crohn disease (Jansson et al., 2009). Therefore, the integration of these omics is necessary for fully detecting microbial community. In a recent study, researchers succeeded to correlate the process of permafrost thawing with microbial composition and functions, using “multi-omics analysis” (Hultman et al., 2015).

## The Connection Between Microbiota and the Human Body

**Figure 2:**
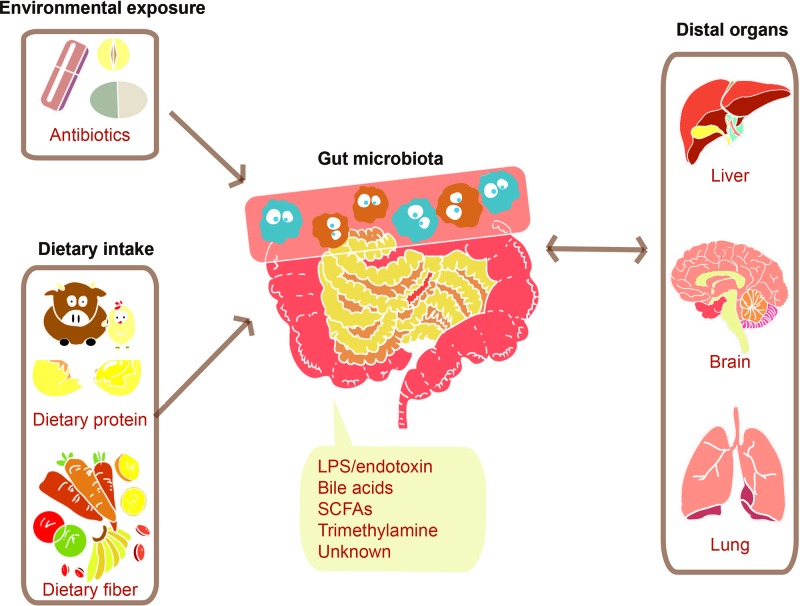
Communications between the gut microbiome and distal organs. Various factors such as environmental exposure and dietary intake can modulate gut microbiota. The change of gut microbiota will bring a certain number of effects on distal organs through signals molecules consisting of their structural components such as lipopolysaccharide (LPS) and their metabolites such as SCFAs.

The dietary intake (Wu et al., 2011; Liu et al., 2018) and environmental exposure such as administration of antibiotics (Pérez-Cobas et al., 2012; Raymond et al., 2016) can largely influence human gut microbiota. The gut microbiota would then respond to these factors, producing signals adjusting human distal organs including liver (Khalsa et al., 2017), brain (Dinan and Cryan, 2017), and lung (Budden et al., 2017), as described in **Figure 2**. Both of microbes’ own structural components and metabolites produced by them can serve as the signal molecules. These signals can affect distal organs metabolism either directly or by signaling through nerves or hormones from the gut (Schroeder and Bäckhed, 2016).

### Gut–Liver Axis

The gut microbiota was confirmed to adjust liver metabolism (Kim et al., 2007; Khalsa et al., 2017). BAs, for example, derived from cholesterol in the liver, can be modified by microbiota in the distal small intestine and colon (Schroeder and Bäckhed, 2016). Primary BAs will be deconjugated by the ileal gut microbiota after they are secreted into the small intestine, which makes them manage to escape the reabsorption and then be subjected to further chemical modification by colonic microbiota (Midtvedt, 1974; Swann et al., 2011). BAs are capable of activating nuclear receptors such as farnesoid X receptor (FXR) and G-protein–coupled receptors (GPCRs), which are associated with host metabolism (Fiorucci et al., 2009). The activation of FXR can suppress the rate-limiting step in BA synthesis through a gut microbiota–liver feedback loop, thus controlling the BA levels (Kim et al., 2007). Additionally, TGR5, one of GPCRs, predominately recognizes secondary BAs, which is associated with increased thermogenesis in brown adipose tissue (Broeders et al., 2015). The adjustment of the gut microbiota on the liver is important, while the response of liver cells is important as well, which can be described using single-cell sequencing. A recent study used single-cell RNA sequencing on T cells from hepatocellular carcinoma patients to identify 11 T-cell subsets with special molecular and functional properties, thus contributing to the prediction of their clinical responses in liver cancer (Zheng et al., 2017).

### Gut–Brain Axis

The association between the brain and other organs depends on complex pathways consisting of the dual autonomic nervous system and endocrine. The gut–brain axis is defined to encompass afferent and efferent neural, endocrine, and nutrient signals between the central nervous system and the gastrointestinal system (Romijn et al., 2008). Several studies have shown that the gut microbiota influences our brain morphology and stress response and even causes the stroke (Schroeder and Bäckhed, 2016) via the gut–brain axis. As for brain morphology, most studies were performed using mice due to the challenges in humans. Through the comparison between germ-free mice and colonized mice, the gut microbiota has been found to cause alterations in the structural integrity of the amygdala and hippocampus (Luczynski et al., 2016). Germ-free mice displayed increased hippocampal neurogenesis and hypermyelination of the prefrontal cortex (Hoban et al., 2016). Moreover, a more permeable blood–brain barrier (BBB) in germ-free mice suggests that the gut microbiota is also capable of modulating the BBB (Braniste et al., 2014). In respect to stress response, *Bifidobacterium longum* was observed to activate the vagus nerve to reduce anxiety-like behavior independently of brain-derived neurotrophic factor (Bercik et al., 2011). Moreover, different community members may have distinct influences on the stress response. For instance, when young germ-free mice with originally elevated stress response were colonized with *Bifidobacterium infantis* at an early developing stage, the stress response was then diminished. But when they were colonized with enteropathogenic *Escherichia coli*, their stress responses were observed to aggravate (Sudo et al., 2004). As to the stroke, 87% are ischemic and caused by interruption of the blood supply to the brain. A study displayed that ischemic brain injury in mice can be reduced by antibiotic-induced alterations in the gut microbiota (Benakis et al., 2016), which provided us with a potential therapeutic method in the future. The characterization of brain cells is important for researchers to further explore the gut–brain axis. Recently, a study performed single-cell sequencing, integrated with multi-omics on the human brain, providing new insights into complex processes in the brain (Lake et al., 2018).

### Gut–Lung Axis

The conception of the gut–lung axis has emerged these years, which still needs more investigations to excavate mechanisms. First, dietary intake can shape both the gut microbiota and the airway microbiota (Marsland et al., 2015). On the one hand, dietary fiber intake leads to an increased level of short-chain fatty acids (SCFAs), which is associated with shifts in both gut microbiota and airway microbiota (Trompette et al., 2014). On the other hand, a high-fat diet has been confirmed to correlate with compositional changes in intestinal microbiota and elevated allergic airway inflammation (Myles et al., 2013). Second, the gut–lung axis contains several interactions among microbiota, metabolites, immune cells, and the lung. Bacterial metabolites such as SCFAs, with the ability to reach other organs *via* the bloodstream, are able to exert their anti-inflammatory properties. Additionally, the microbial seeding from the intestinal microbiota into the airways makes these bacteria able to act on local immune cells to shape their responses (Marsland et al., 2015). Moreover, migrating immune cells are capable of acquiring information directly from microbiota and the concomitant local cytokine response to adjust inflammatory response, which shapes immune responses at distal sites such as the lung (Trompette et al., 2014; Budden et al., 2017). Scientists have correlated allergic asthma, one of the lung diseases, with the gut microbiota. A study displayed that a fecal transplant from a child at risk of asthma into germ-free mice resulted in severe lung inflammation after challenge with ovalbumin (Arrieta et al., 2015). Moreover, another study showed that the impacts by recurrent antibiotic treatment on the diversity of the microbiota early in life (Fouhy et al., 2012) have been confirmed to strongly correlate with the development of an asthmatic phenotype later in life (Fanaro et al., 2003). There are still a certain number of unknown mechanisms in the gut–lung axis, which provides us with a lot of potential therapeutic methods against lung diseases.

## Microbiota and Clinical Medicine

### Gastrointestinal Disease

**Figure 3:**
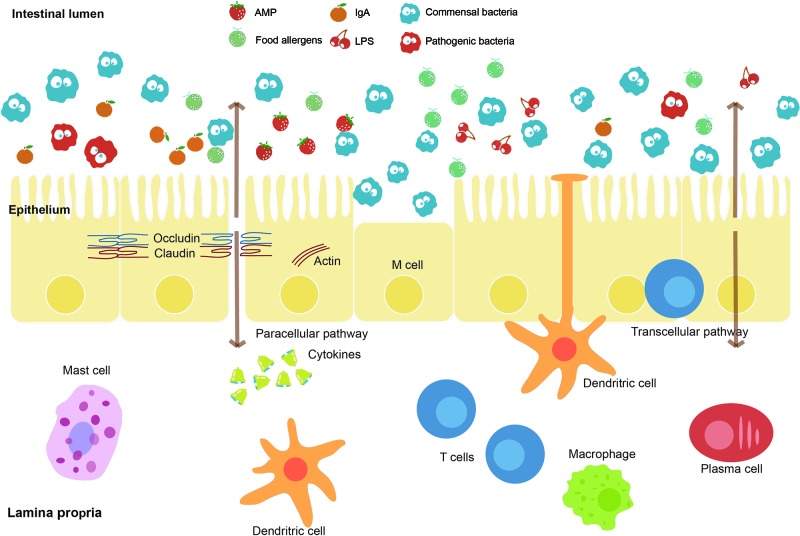
Intestinal barrier and affecting factors. The intestinal barrier, as an essential barrier against harmful pathogens and substances in the intestine, mainly consists of the mucus layer, the epithelial layer, and the underlying lamina propria. The intestinal lumen contains antimicrobial peptides (AMPs), secreted IgA, and commensal bacteria, which prevent the colonization of pathogens. A mucus layer covers the intestinal surfaces as a physical barrier. The epithelium is composed of a single layer of cells sealed by tight junction proteins such as occludin and claudin inhibiting paracellular passage. M cells and intraepithelial lymphocytes are also contained in this layer. The lamina propria harbors lots of immune cells. Factors including food allergens, lipopolysaccharides (LPS), and pathogenic bacteria such as EPEC effect on the intestinal barrier function.

The intestine is a critical organ in the human’s body, whose functions involve the uptake of nutrients and water. The intestinal barrier (**Figure 3**), as the essential barrier of the intestine, prevents the transfer of harmful substances and pathogens. Pathogenic bacteria may cause the disruption of this barrier resulting in increased intestinal permeability. Enteropathogenic E. coli (EPEC), for instance, causes a loss of enterocyte microvilli and the formation of a raised pedestal structure for firm bacterial attachment (Lapointe et al., 2009). In addition, enterohemorrhagic E. coli also possesses an attaching and effacement locus but with less profound effects on the barrier (Kaper and Nataro, 2004). Moreover, enteroaggregative E. coli and enterotoxigenic E. coli can cause diarrhea through effects on chloride secretion in the intestinal epithelium (Dubreuil, 2012). The single-cell sequencing helps to identify the pathogenic microbes at the intestinal lumen. The main antibody isotype named immunoglobulin A (IgA), which is produced at mucosal surfaces, can bind those pathogenic microbes in the intestinal lumen. The cell sorting then uses a fluorescent anti-IgA antibody, followed by 16S rDNA sequencing to identify the isolated pathogenic microbes (Palm et al., 2014). Furthermore, metagenomic sequencing can also be performed on these isolated microbes to identify the basis of immunogenic differences between and within microbes. Similarly, the elevated IgG coating of gut bacteria has also been observed in patients with sepsis and Crohn disease system (Zeng et al., 2016). Therefore, the single-cell sequencing is a promising method to correlate microbes with host immune response for precision medicine (Tolonen and Xavier, 2017).

### Thrombosis

The risk of thrombosis has been observed to be correlated with the plasma levels of trimethylamine (TMA)–N-oxide (TMAO) in humans (Zhu et al., 2016). Especially, the gut microbiome is critically involved in the generation of TMAO (Tang et al., 2013). The gut microbiome can process certain dietary nutrients such as phosphatidylcholine, choline, and carnitine specifically to procedure TMA, which is absorbed in the gut and converted in the liver to TMAO by hepatic flavin-containing monooxygenases (Tilg, 2016). In humans, foods such as meat and eggs have been associated with an increased risk of major cardiovascular events in patients with proven coronary heart disease (Tang et al., 2013). In addition, administration of antibiotics can markedly reduce the plasma levels of TMAO.

### Hepatitis B Virus

Hepatitis B virus (HBV), as one of the most common infectious agents worldwide, has been associated with the gut microbiome (Chou et al., 2015). Scientists have found that viral clearance heavily depends on the age of exposure. According to the control experiments of adult and young mice, the results showed an immune-tolerating pathway to HBV that prevailed in young mice with immature gut microbiota. After the establishment of gut bacteria, the mature gut microbiota in adult mice stimulated liver immunity, resulting in rapid HBV clearance (Chou et al., 2015). Therefore, full understanding of the interaction of virus–host may help us with the therapy for HBV. The single-cell sequencing can serve as a powerful method to explore the virus–host interaction (Labonte et al., 2015).

### Depression

Depressive episodes correlate with dysregulation of the hypothalamic–pituitary–adrenal (HPA) axis (Barden, 2004) and resolution of depressive systems with normalization of the HPA axis (Heuser et al., 1996; Nickel et al., 2003). The gut microbiota has been confirmed to play a part in both the programming of the HPA axis early in life and stress reactivity over the life span (Foster and Neufeld, 2013). The stress response system is functionally immature at birth and then develops throughout the postnatal period, which coincides with the intestinal bacterial colonization. Stress can increase intestinal permeability, providing bacteria with an opportunity to translocate across the intestinal mucosa and directly access both immune cells and neuronal cells of the enteric nervous system (Gareau et al., 2008; Teitelbaum et al., 2008).

### AIDS

The gut microbiota has been recently observed to be associated with human immunodeficiency virus (HIV) disease progression (Vujkovic-Cvijin et al., 2013). Scientists identified a dysbiotic mucosal-adherent community enriched in Proteobacteria and depleted of Bacteroidia members that were associated with markers of mucosal immune disruption, T-cell activation, and chronic inflammation in HIV-infected subjects. This dysbiotic community was evident among HIV-infected subjects undergoing highly active antiretroviral therapy (Vujkovic-Cvijin et al., 2013). Furthermore, the extent of dysbiosis correlated with two established markers of disease progression including the activity of the kynurenine pathway of tryptophan catabolism and plasma concentrations of the inflammatory cytokine interleukin 6 (Vujkovic-Cvijin et al., 2013). Hence, a link between mucosal-adherent colonic bacteria and immunopathogenesis during progressive HIV infection deserves better investigations.

### Cancer

Gut microbes have been reported to be correlated with a certain number of cancers related to human stomach (*Helicobacter pylori*), liver (*Opisthorchis viverrini*, *Clonorchis sinensis*), and bladder (*Schistosoma haematobium*) (Bhatt et al., 2017). *H. pylori* infections, for instance, can lead to gastritis and gastric ulcers (Marshall et al., 1984), which is considered as the precursor of gastric cancer. Nevertheless, *H. pylori* was also observed to protect against esophageal adenocarcinoma, by influencing stomach pH and ameliorating acid reflux (Vaezi et al., 2000). Hence, owing to the participation of microbes in multiple biological processes, the oncogenicity of microbes should be discussed and determined by multi-omics approaches.

## The Trend of Big-Data Mining for Microbiome

In the past, owing to limitations in abilities to obtain and process microbial big data, scientists were not able to obtain a full understanding of the microbiota. Neither the sequencing technologies nor the analysis tools can meet the high dimensional complicacy of the intestinal microbiota. Nowadays, the high-throughput sequencing technologies, such as MDA (Dean et al., 2002) for single-cell sequencing, and numerous statistical analysis tools, such as QIIME for 16S sequencing data (Caporaso et al., 2010) and MetaPhlAn (Segata et al., 2012) for metagenomics data, make it possible to unveil the microbiota from various perspectives. The integration of the current sequencing methods would be necessary to conduct a comprehensive study on microbiota in the future. First, the taxonomic information at various levels can be obtained by amplicon sequencing and metagenomic sequencing. Second, the functional annotation can be predicted by metagenomics and confirmed by the multi-omics including metagenome, metatranscriptome, metaproteome, and metabolome. Third, the connection between functions and phylogeny of a single microbe cell can be established by single-cell sequencing. Finally, the interactions between all chromosomes can be detected by Hi-C sequencing. The integration of these methods can answer the questions “who is there,” “what are they doing,” and “how are they doing” from a macroscopic level of overall microbial composition and microscopic level of single microbe cell and even the single chromosome. The comprehensive analysis of big data, followed by strict *in vivo* and *in vitro* experiments, is required to determine the causality of clinical diseases by microbes for specific medicine. Moreover, a standard pipeline for the integration of these methods proposed in the future can produce a huge amount of data sets. The big-data sets across continents provide the spatial characteristics, and the big-data sets in the long-term investigations provide the characteristics at time scale.

## Author Contributions

KN conceived the review framework. MC and LC conducted the literature review. MC made the figure illustration. MC and LC wrote the manuscript. KN reviewed and revised the manuscript. 

## Funding

This work was partially supported by the National Key R&D Program of China (grant 2018YFC0910502) and the National Natural Science Foundation of China (grants 61103167, 31271410, and 31671374).

## Conflict of Interest

The authors declare that the research was conducted in the absence of any commercial or financial relationships that could be construed as a potential conflict of interest. 

The handling editor declared a past co-authorship with one of the authors KN.
